# Supplementary figures and images for: Cow dung putrefaction via vermicomposting using Eisenia fetida and its influence on seed sprouting and vegetative growth of Viola wittrockiana (pansy)
Source: PLoS One. 2023 Feb 17;18(2):e0279828. doi: 10.1371/journal.pone.0279828 (PMC9937483; doi:10.1371/journal.pone.0279828)

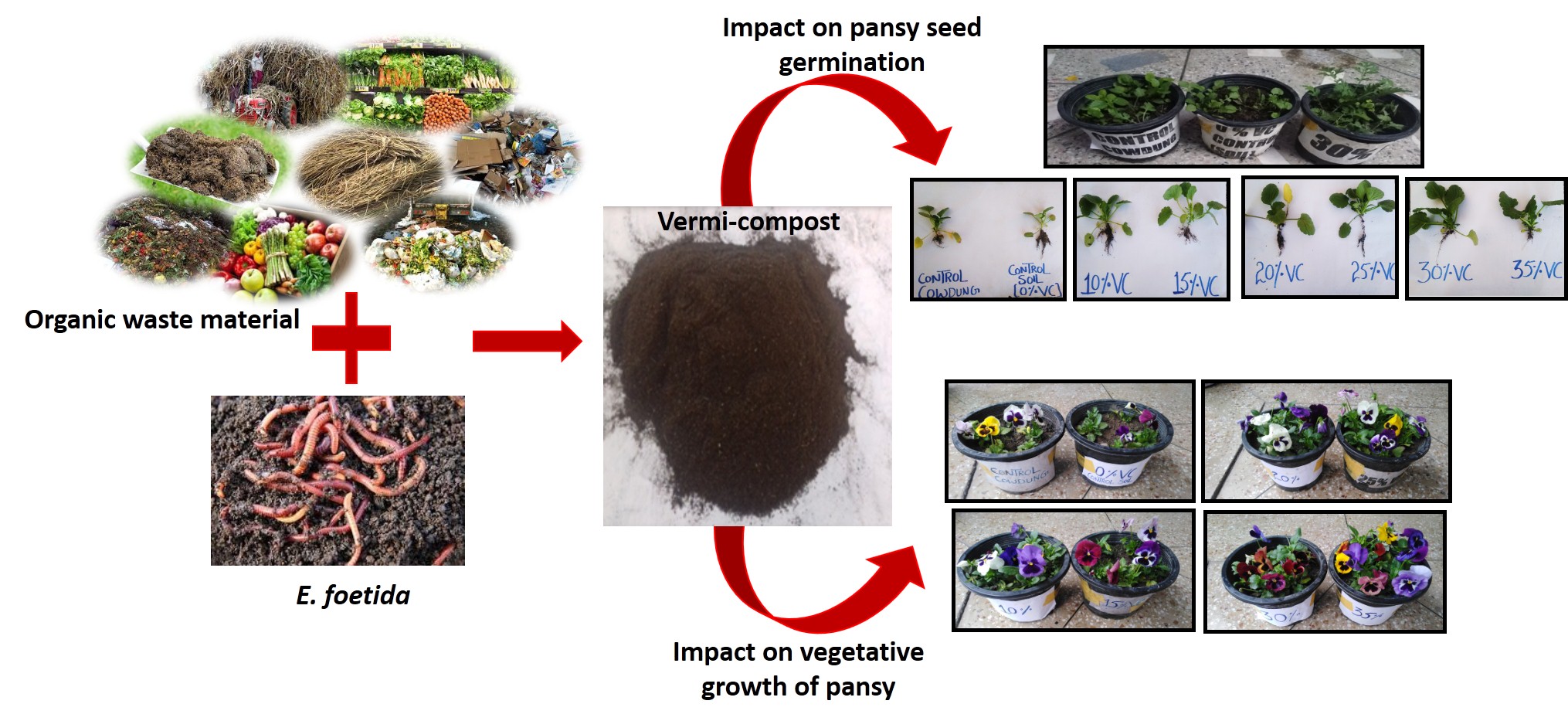

Supplement: S1 Graphical abstract — (JPG) [file pone.0279828.s001.jpg]
