# Supplementary material for: Cow dung putrefaction via vermicomposting using Eisenia fetida and its influence on seed sprouting and vegetative growth of Viola wittrockiana (pansy)
Source: PLoS One. 2023 Feb 17;18(2):e0279828. doi: 10.1371/journal.pone.0279828 (PMC9937483; doi:10.1371/journal.pone.0279828)

PLOS ONE

Cow dung putrefaction via vermicomposting using Esinea fetida and its impact on seed germination and plant growth parameters of Viola wittrockiana (pansy)

--Manuscript Draft--

**Manuscript Number:**	PONE-D-22-12324
**Article Type:**	Research Article
**Full Title:**	Cow dung putrefaction via vermicomposting using Esinea fetida and its impact on seed germination and plant growth parameters of Viola wittrockiana (pansy)
**Short Title:**	IMPACT OF VERMICOMPOST ON Viola wittrockiana (pansy)
**Corresponding Author:**	Saiqa Andleeb<br>The University of Azad Jammu & Kashmir<br>Muzaffarabad, ak PAKISTAN
**Keywords:**	Vermicompost, pansy, seed germination, germination media, cow dung, flowering parameters, plant growth parameters.
**Abstract:**	Nowadays, vermicompost is considered a promising organic fertilizer alternative to chemical fertilizers in agriculture and horticulture. Therefore, current research aimed to improve soil health via vermicompost utilization and to evaluate the impact of vermicompost and organic manure (cow dung) on seed germination, seedlings, and vegetative growth parameters of Viola x wittrokiana (pansy). In the current study, vermicompost was produced using various organic waste materials via Esinea fetida . Physio-chemical parameters of vermicompost and organic manure were recorded. Potting experiment was designed, germination medium containing soil, sand, and various concentrations of vermicomposts were prepared as T 0 (Sand+Soil), Tcc (Sand+Soil+Cow dung), 10% VC (Sand+Soil+0.1 kg VC), 15% VC (Sand+Soil+0.15 kg VC), 20% VC (Sand+Soil+0.2 kg VC), 25% VC (Sand+Soil+0.25 kg VC), 30% VC (Sand+Soil+0.3 kg VC), and 35% VC (Sand+Soil+0.35 kg VC). Seed germination, seedling, vegetative plant growth and flowering parameters were evaluated in different germination media. Pre and post physio-chemical parameters of germination media were also recorded to check their stability and quality. Results showed that 30% VC germination media was effective dose for early seed germination initiation and for all seed germination parameters of pansy. However, the best vegetative plant growth and flowering parameters of pansy plants occurred at 35% VC after seedlings transplantation. Findings showed the significant impact of vermicompost not only on the seed germination but also on the seedling developments and could be used as best suited germination and growing media. These results indicate that vermicompost can be exploited as a potent bio-fertilizer for ornamental plant production.
**Order of Authors:**	Irsa Shafique
	Saiqa Andleeb
	Farrukh Hassan
	Shaukat Ali
	Tauseef Tabassum
	Tariq sultan
	Muhammad Almas Abbasi
**Additional Information:**	
**Question**	**Response**
**Financial Disclosure**<br><br>Enter a financial disclosure statement that describes the sources of funding for the work included in this submission. Review	Saiqa Andleeb<br>TDF-02-06<br>Higher Education Commission Pakistan<br>The funders had no role in study design, data collection and analysis, decision to publish, or preparation of the manuscript.

Powered by Editorial Manager® and ProduXion Manager® from Aries Systems Corporation

the submission guidelines for detailed requirements. View published research articles from *PLOS ONE* for specific examples.<br><br>This statement is required for submission and **will appear in the published article** if the submission is accepted. Please make sure it is accurate.<br><br>**Unfunded studies**<br>Enter: *The author(s) received no specific funding for this work.*<br><br>**Funded studies**<br>Enter a statement with the following details:<br>• Initials of the authors who received each award<br>• Grant numbers awarded to each author<br>• The full name of each funder<br>• URL of each funder website<br>• Did the sponsors or funders play any role in the study design, data collection and analysis, decision to publish, or preparation of the manuscript?<br>• **NO** - Include this sentence at the end of your statement: *The funders had no role in study design, data collection and analysis, decision to publish, or preparation of the manuscript.*<br>• **YES** - Specify the role(s) played.<br><br>* typeset	
**Competing Interests**<br><br>Use the instructions below to enter a competing interest statement for this submission. On behalf of all authors, disclose any competing interests that could be perceived to bias this work—acknowledging all financial support and any other relevant financial or non-financial competing interests.<br><br>This statement is required for submission and **will appear in the published article** if the submission is accepted. Please make sure it is accurate and that any funding sources listed in your Funding Information later in the submission form are also	The authors have declared that no competing interests exist.

declared in your Financial Disclosure statement.<br><br>View published research articles from *PLOS ONE* for specific examples.<br><br>**NO authors have competing interests**<br><br>Enter: *The authors have declared that no competing interests exist.*<br><br>**Authors with competing interests**<br><br>Enter competing interest details beginning with this statement:<br><br>*I have read the journal's policy and the authors of this manuscript have the following competing interests: [insert competing interests here]*<br><br>* typeset	
**Ethics Statement**<br><br>Enter an ethics statement for this submission. This statement is required if the study involved:<br><br>• Human participants<br>• Human specimens or tissue<br>• Vertebrate animals or cephalopods<br>• Vertebrate embryos or tissues<br>• Field research<br><br>Write "N/A" if the submission does not require an ethics statement.<br><br>General guidance is provided below. Consult the submission guidelines for detailed instructions. **Make sure that all information entered here is included in the Methods section of the manuscript.**	N/A

**Format for specific study types**<br><br>**Human Subject Research (involving human participants and/or tissue)**<br>• Give the name of the institutional review board or ethics committee that approved the study<br>• Include the approval number and/or a statement indicating approval of this research<br>• Indicate the form of consent obtained (written/oral) or the reason that consent was not obtained (e.g. the data were analyzed anonymously)<br><br>**Animal Research (involving vertebrate animals, embryos or tissues)**<br>• Provide the name of the Institutional Animal Care and Use Committee (IACUC) or other relevant ethics board that reviewed the study protocol, and indicate whether they approved this research or granted a formal waiver of ethical approval<br>• Include an approval number if one was obtained<br>• If the study involved *non-human primates*, add *additional details* about animal welfare and steps taken to ameliorate suffering<br>• If anesthesia, euthanasia, or any kind of animal sacrifice is part of the study, include briefly which substances and/or methods were applied<br><br>**Field Research**<br><br>Include the following details if this study involves the collection of plant, animal, or other materials from a natural setting:<br>• Field permit number<br>• Name of the institution or relevant body that granted permission	
**Data Availability**<br><br>Authors are required to make all data underlying the findings described fully available, without restriction, and from the time of publication. PLOS allows rare exceptions to address legal and ethical concerns. See the PLOS Data Policy and FAQ for detailed information.	Yes - all data are fully available without restriction

A Data Availability Statement describing where the data can be found is required at submission. Your answers to this question constitute the Data Availability Statement and **will be published in the article**, if accepted.<br><br>**Important:** Stating 'data available on request from the author' is not sufficient. If your data are only available upon request, select 'No' for the first question and explain your exceptional situation in the text box.<br><br>Do the authors confirm that all data underlying the findings described in their manuscript are fully available without restriction?	
**Describe where the data may be found in full sentences. If you are copying our sample text, replace any instances of XXX with the appropriate details.**<br><br>• If the data are **held or will be held in a public repository**, include URLs, accession numbers or DOIs. If this information will only be available after acceptance, indicate this by ticking the box below. For example: *All XXX files are available from the XXX database (accession number(s) XXX, XXX.).*<br>• If the data are all contained **within the manuscript and/or Supporting Information files**, enter the following: *All relevant data are within the manuscript and its Supporting Information files.*<br>• If neither of these applies but you are able to provide **details of access elsewhere**, with or without limitations, please do so. For example:<br><br>*Data cannot be shared publicly because of [XXX]. Data are available from the XXX Institutional Data Access / Ethics Committee (contact via XXX) for researchers who meet the criteria for access to confidential data.*<br><br>*The data underlying the results presented in the study are available from (include the name of the third party*	All relevant data are within the manuscript and/OR Supporting Information files

and contact information or URL).<br>• This text is appropriate if the data are owned by a third party and authors do not have permission to share the data.<br><br>* typeset	
Additional data availability information:	

Powered by Editorial Manager® and ProduXion Manager® from Aries Systems Corporation

Manuscript Click here to access/download;Manuscript;Shafique et al., 2022.doc

Cow dung putrefaction *via* vermicomposting using *Esinea fetida* and its impact on seed germination and plant growth parameters of *Viola wittrockiana* (pansy)

Irsa Shafique[1], Saiqa Andleeb[1*], Farrukh Hassan[2], Shaukat Ali[3], Tauseef Tabasum[4], Tariq Sultan[4], Mohammad Almas Abbasi[5]

[1]Microbial Biotechnology and Vermi-technology Laboratory, Vermi-tech Unit, Department of Zoology, University of Azad Jammu and Kashmir, Muzaffarabad, 13100, Pakistan

[2]First Biotech LLC, Lahore

[3]Department of Zoology, GC University, Lahore, Pakistan

[4]Land Resources Research Institute, Pakistan Agricultural Research Council, Islamabad, Pakistan

[5]Department of Agriculture, Muzaffarabad, Azad Jammu and Kashmir, 13100, Pakistan

*Corresponding author: drsaiqa@gmail.com , drsaiqa@ajku.edu.pk

ABSTRACT

Nowadays, vermicompost is considered a promising organic fertilizer alternative to chemical fertilizers in agriculture and horticulture. Therefore, current research aimed to improve soil health *via* vermicompost utilization and to evaluate the impact of vermicompost and organic manure (cow dung) on seed germination, seedlings, and vegetative growth parameters of *Viola x wittrokiana* (pansy). In the current study, vermicompost was produced using various organic waste materials *via Esinea fetida*. Physio-chemical parameters of vermicompost and organic manure were recorded. Potting experiment was designed, germination medium containing soil, sand, and various concentrations of vermicomposts were prepared as T_0 (Sand+Soil), Tcc (Sand+Soil+Cow dung), 10% VC (Sand+Soil+0.1 kg VC), 15% VC (Sand+Soil+0.15 kg VC), 20% VC (Sand+Soil+0.2 kg VC), 25% VC (Sand+Soil+0.25 kg VC), 30% VC (Sand+Soil+0.3 kg VC), and 35% VC (Sand+Soil+0.35 kg VC). Seed germination, seedling, vegetative plant growth and flowering parameters were evaluated in different germination media. Pre and post physio-chemical parameters of germination media were also recorded to check their stability and quality. Results showed that 30% VC germination media was effective dose for early seed germination initiation and for all seed germination parameters of pansy. However, the best vegetative plant growth and flowering parameters of pansy plants occurred at 35% VC after seedlings transplantation. Findings showed the significant impact of vermicompost not only on the seed germination but also on the seedling developments and could be used as best suited germination and growing media. These results indicate that vermicompost can be exploited as a potent bio-fertilizer for ornamental plant production.

Keywords: Vermicompost, pansy, seed germination, germination media, cow dung, flowering parameters, plant growth parameters.

INTRODUCTION

Sustainable agriculture can be defined as a set of practices that conserve resources and the environment without compromising human needs, and the use of organic fertilizers such as animal manure has been indicated as one of its main pillars (Tilman et al. 2002). Animal manure is a valuable resource as a soil fertilizer because it provides large amounts of macro-and micronutrients for crop growth and is a low-cost, environmentally-friendly alternative to mineral fertilizers. However, the use of manure in agriculture is being abandoned because of increasing transportation costs and environmental problems associated with the indiscriminate and inappropriately-timed application to agricultural fields (Hutchison et al. 2005). Processing of this waste material through controlled bio-oxidation processes, such as composting, reduces the environmental risk by transforming the material into a safer and more stable product suitable for application to soil (Lazcano et al. 2008), and also reduces the transportation costs because of the significant reduction in the water content of the raw organic matter. Composted materials are therefore gaining acceptance as organic fertilizers in sustainable agriculture, and there has been a considerable increase in research dedicated to the study of the effects of compost-like materials on soil properties and plant growth. Although most of the research has been focused on the impacts of some widely-known organic amendments

such as animal manures, green manures, and compost, research into other alternative organic amendments such as vermicompost is gaining importance (Edwards et al. 2004).

Vermicomposting is a biological process used to convert complex organic wastes into a stabilized nutrient-rich fertilizer called vermi-compost. Vermi-composts are peat-like materials containing plant growth hormones and nutrients in plant-available forms (Lim et al. 2016). In vermicomposting process, earthworms and microbes of their gut degrades organic waste and forms a stable and mature product (Bhat et al. 2013; Lim et al. 2016). Organic waste recycling and its use as a soil conditioner is the attractive method for waste management. The application of organic manures produced from organic wastes by vermicomposting is one of the ways to maintain soil fertility and reduce soil pollution (Ostos et al. 2008; Bhat et al. 2016a). Different species of earthworms are used in vermicomposting such as *Eisenia fetida*, *Perionyx excavates,* and *Eudrilus eugeniae,* etc (Rajendran and Thivyatharsan 2014). In Pakistan, *Eisenia fetida* is the commonly used earthworm species for vermicomposting.

The use of compost in horticulture has occasionally been shown to be limited by the high electrical conductivity and the excessively high amount of certain ions that cause phytotoxicity (Garcia-Gomez et al. 2002), as a consequence of the chemical properties of the initial waste and /or inadequate composting procedures. These adverse effects, although possible, are less likely to occur when vermicompost is used as a potting amendment (Chaoui et al. 2003). Among their superior chemical attributes, Edwards and Burrows (1988) reported that vermicomposts, especially those from animal waste sources, usually contained more mineral elements than commercial plant growth media, and many of these elements were changed to forms more that could be readily taken up by the plants, such as nitrates, exchangeable phosphorus, and soluble potassium, calcium, and magnesium. Orozco et al. (1996) reported that coffee pulp, increased the availability of nutrients such as phosphorus, calcium, and magnesium, after processing by *Eisenia fetida*. Recently there has been an increasing pressure on horticultural producers to increase water and nutrient use efficiency due to environmental concerns, and organic amendments are starting to be considered in conventional horticultural practices (Marfa et al. 2002). Therefore, it would appear that the addition of small amounts of vermicompost into the potting substrates, would allow the commercial ornamental growers to easily increase both the production and quality of their crops. According to this, we evaluated the feasibility of incorporating vermicompost into the potting media of commercially- grown pansies (*Viola wittrockiana*).

The garden pansy (*Viola wittrockian*a) called ‘the flower of all seasons’ is cultivated on the largest scale in the world as a bedded and pot plant (Startek 2003). Pansies typically display large showy face markings. Modern horticulturists have developed a wide range of pansy flower colors and bi-colors including yellow, gold, orange, purple, violet, red, white, and even near-black (very dark purple). Apart from being used for decorative purposes, both the leaves and flowers of pansies and violas are edible and high in vitamins A and C. The flowers impart a strong flavor and have been used to make syrup, flavored honey, and salads. Both the leaves and flowers can be used as a garnish, such as on cold fruit or cream soups (Bohdanowicz et al. 2012). They have medicinal importance as well, they are used as anti-allergen, very helpful for cold and flu as it soothes coughs and sore throat, and is also helpful in treating fevers, bronchitis, asthma, and whooping cough (Lewis and Lewis 2003). For many years’ pansies have been cultivated in peat substrate supplemented with traditional and slow-release fertilizers. (Zawadzinska and Janicka 2007). Startek et al. (2006a) found that coconut media have a favorable effect on both the pansy emergence and further growth. Due to limited peat resources and great costs of media import, it is advisable to search for alternate media, rich in nutrients with good physical properties. Thus in the current study, vermicompost was used to screen its impact on seed germination, seedling, and plant growth parameters and flowering of *Viola wittrockian*a.

MATERIALS AND METHODS

The experiment was carried out in the Vermi-tech Unit, Department of Zoology, University of Azad Jammu and Kashmir, Muzaffarabad, Pakistan. The seeds of *Viola wittrokiana* (pansy) were purchased from Evergreen nursery Islamabad, Pakistan, and potting experiments were conducted in open house conditions.

Waste material and earthworm collection

Animal wastes (cow dung) were collected from local residential houses of Muzaffarabad, Azad Jammu, and Kashmir, Pakistan. The cow dung was air-dried for 10 to 15 days to remove the various associated organisms (beetles, spiders, snails, etc), noxious gases, and chopped into small pieces (Bhatnagar and Palta, 1996). The organic waste materials such as grass clippings, raw vegetables and fruits (their peels as well), maize straw, coconut peel, rice straw, eggshells, raw papers, and dry leaves were also collected from the residential houses and local market of Muzaffarabad, Azad Jammu and Kashmir, Pakistan. Raw waste products such as vegetables and fruits were pre-

digested before use. Fresh spinach or green vegetables were used for vermi-culturing. Waste papers were dipped in water for 24 hrs before bedding. Locally available earthworms *E. fetida* were collected from the local area (Mahajar camp) of Muzaffarabad, Azad Jammu, and Kashmir, Pakistan, in sealing bags or plastic bottles.

Experimental design for vermicompost production

The vermicomposting experiment was carried out in cemented pits (10 x 5 feet) for 3 months. A worm bed was constructed by using moisten papers/shredded twigs/grass clippings, varying according to what is available locally. Feed matter like wheat straw, cow dung, green leaves, crushed eggshells, brown leaves, and tea bags was given to earthworms. Cow manure was mixed with the pre-digested substrate in a 3: 1 ratio. At the beginning of vermicomposting 100 to 200 *E. fetida* were added to the top of the pit. To protect the worm population from predators such as birds, rats, cockroaches, and ants the pre-compost material was covered by either banana leaves, wire mesh, or cloth cover. The vermicomposting process was checked once a week and thorough mixing of all substrate material was done to ensure the availability of all feeding materials are required for vermi-culturing. The temperature and moisture contents were maintained by the sprinkling of water. After 3 months, the change in the texture, appearance, color, electrical conductivity, temperature, moisture, and the earthy smell was observed as the indicators of the formation of vermicompost. Finally, composting material was passed through a 2 mm sieve and used for measuring the desirable parameters.

Biomass and reproduction (number) of earthworms

At the end of process the total biomass of *E. fetida*, the total number of juveniles, adults, and cocoons were recorded. The fecundity rate was calculated as number of earthworms/total number of earthworms used x 100.

Physio-chemical analysis of vermicompost and cow dung

All physio-chemical parameters were measured at the Soil testing laboratory of Soil Research institute, Gojra, AJ&K, Muzaffarabad, Pakistan. Physical parameters of vermicompost and cow dung such as color, texture appearance, color, electrical conductivity (EC), temperature, moisture, and the smell was recorded. To accurately measure the physical parameters affecting composting, standard methods were used for the chemical analysis of each parameter (Apha et al. 2012). The total carbon (C%), nitrogen (N%) were measured using the Walkley-Black method whereas potassium (K%) and phosphorus (P%) levels in the vermicompost and cow dung were measured by ammonium acetate method and modified Olsen method, respectively (Watanabe and Olsen, 1965; Olsen and Sommers, 1982). In addition, the concentration of trace metals such as manganese, calcium, sulfates, copper, magnesium, zinc, and iron were measured through atomic absorption spectrometry (Naouni et al. 2011; Shahmansouri et al. 2005).

Experimental setup/design

The plastic pots of 1 kg capacity were used to evaluate the effect of organic manure (cow dung) and prepared vermicompost on seed germination and plant growth parameters (length of shoot, number of leaves and their measurements, root length etc.) of pansy. The pots were individually filled with the germination medium contain soil, sand, and various concentrations of cow dung based vermicompost. The treatment details are given as: To-control (0.33 kg sand and 0.67 kg soil), Tcc-control (0.33 kg sand + 0.32 kg soil + 0.35 kg cow dung), 10% VC (0.33 kg sand + 0.57 kg soil + 0.1 kg VC), 15% VC (0.33 kg sand + 0.52 kg soil + 0.15 kg VC), 20% VC (0.33 kg sand + 0.47 kg soil + 0.2 kg VC), 25% VC (0.33 kg sand + 0.42 kg soil + 0.25 kg VC), 30% VC (0.33 kg sand + 0.37 kg soil + 0.3 kg VC), and 35% VC (0.33 kg sand + 0.32 kg soil + 0.35 kg VC).

Pre and post-physio-chemical analysis of germination media

To check the stability and quality of germination media before sowing and after harvesting of pansy seedlings, physio-chemical parameters pH, EC, total carbon (%), nitrogen (%), potassium (%), and phosphorus (%) were measured using protocols as mentioned above.

Seed sowing and maintenance

Each treatment was used in triplicates, 8 seeds were manually sown with equal spacing between the seeds at a uniform depth of 3 cm in each of 24 pots having a capacity of 1 kg and were watered thoroughly each day (morning) so that all the treatments received the same amount of irrigation. The pots were arranged randomly in the natural sunlight with the positions re-randomized every week. Daily observations were made on radicle emergence. Proper care was given to avoid damage to the seed.

Observations of seed germination and seedling growth parameters

The sowing day was considered as the first day and the effect of germination media on seed germination was observed in the morning every day. The total number of germinated seeds was counted every day. Germination percentage was recorded after 30 days of seedlings as: germination % = Number of seedlings/total number of seeds x 100. Initiation of seed germination (First plumule emerged), Speed of germination (n1/d1+n2/d2+n3/d3+----- Where, n = number of germinated seeds, and d= number of days), Mean germination Time (MGT= n1 x d1 + n2 x d2 + n3 x d3 + --------/ Total number of days: Where, n= number of germinated seed, and d = number of days), Mean daily germination (MDG = Total number of germinated seeds/ Total number of days.), Peak Value (PV= Highest seed germinated/ Number of days.), Germination values (= PV X MDG), and completion of germination (The day from which no further germination occurred) were also calculated (Czabator 1962; Ellis and Roberts 1981; ISTA 1999). After 30 days of the experiment, seedling growth parameters such as shoot length (cm), number of leaves, the diameter of leaves (cm), length and area of leaves (cm), the whole length of seedling (cm), and root length (cm), were also recorded.

Transplantation of pansy plants

The pansy seedlings were transplanted after 30 days in the pots (7 kg) containing germination medium in the mid of November when the climatic temperature (10–15 °C) is best for their growth. To study the impact of vermicompost on plant growth parameters same germination media (%) were used. In each 7 kg pot, three seedlings were transplanted. A potting experiment was conducted in an open house having no other environmental control with a maximum range of temperature from 10-15 °C, a minimum range of temperature 5-10 °C with bright sunlight, and active photoperiod of 7-8 h. Plants were harvested at the yield stage. Different plant growth parameters like plant height (cm), root volume, the diameter of leaves (cm), number of leaves, initiation of flowering, number of open flowers, number of floral buds, the diameter of flower (cm), dry and fresh weight of flowers (g) were investigated. Before transplantation and after harvesting of pansy plants, physio-chemical parameters pH, electrical conductivity, total organic matter (%), total carbon (%), nitrogen (%), potassium (%) and phosphorus (%) were measured using protocols as mentioned above.

Statistical analysis

All experiments were carried out in triplicate. Data were expressed as Mean±Standard Error. The statistical significance for various treatments was evaluated by one-way analysis of variance (ANOVA) using Graph Pad Prism for Windows (version 5.03) and also used to plot graphs with error bars of standard errors of the means (SEM). ‘a’ represents a significant change among Tcc and VC treatment groups and ‘b’ represents a significant change among To and VC treatment groups. Statistical icons: a=$p\leq0.05$; aa=$p\leq0.01$; and aaa=$p\leq0.001$

RESULTS

Physical analysis of vermicompost and cow dung

Vermicompost has been produced on large scale at Vermi-tech Unit, Department of Zoology, University of Azad Jammu and Kashmir, Muzaffarabad using various organic waste materials and *E. fetida* earthworm species. After 3 months all the waste material was completely converted into humus-like material with high porosity (vermi-compost) *via E. fetida*. Physical properties such as color, smell, appearance, temperature, pH, electrical conductivity, and moisture contents were recorded from both the organic manure (Cow dung) and vermicompost at the beginning and end of the experimental work. Physical properties of cow dung were recorded as greenish to dark brown color, pasty and the pile of shaving cream-like appearance, pungent or rotten egg-like smell, 55-60°C temperature, 8.7 pH, 3.52 mS/cm EC, and 18 % moisture contents, respectively. The physical properties of finely prepared vermicompost were recorded as blackish-brown in color, fine grain, and porous in appearance with an earthy smell, 25 °C temperature, 7.8 pH, 2.82 mS/cm EC, and 25 % moisture contents, respectively.

Chemical analysis of vermicompost and cow dung

The current research revealed that vermicompost possessed a significantly ($p\leq0.001$) high level of nutrients such as nitrogen, phosphorous, potassium, calcium, and magnesium compared to cow dung manure. The total organic carbon and organic matter were significantly ($p\leq0.001$) reduced in vermicompost (25.2% and 43%) compared to cow dung (50.4 % and 86 %). On the other hand, the percentage of nitrogen (N), phosphorus (P), and potassium (K) were increased in vermicompost (2.28%, 0.030%, and 0.033%) compared to cow dung (1.86%, 0.0098%, and 0.020), respectively. The cation exchange capacity of vermicompost was greater (72.48 me/100g) compared to cow

dung, 62.30 me/100g. The Carbon to Nitrogen ratio (C: N) was reduced in vermicompost (11:1) paralleled to cow dung (26:1), respectively. The level of trace metals such as sulfates (0.55 %), iron (1.3313 %), zinc (0.110 %), manganese (0.2038 %), magnesium (0.78 %), Copper (0.0048 %) and calcium (1.35 %) were also increased in vermicompost compared to the cow dung such as sulfates (0.35 %), iron (1.1690%), zinc (0.0012 %), manganese (0.0414 %), magnesium (0.25 %), Copper (0.0017 %), and calcium (1.0 %).

Physio-chemical analysis of soil and sand before sowing

Results revealed that soil used for marigold seed germination before sowing was sandy loam in texture, the brown color having pH 7.64, and electrical conductivity 2.0 mS/cm. Chemically the soil consisted of 0.0014% potassium, 0.61% organic carbon, 1.049% organic matter, 0.52% nitrogen and 0.0072% phosphorus. Similarly, the chemical analysis of sand used for the experiment showed 2.64% organic matter, 1.53% organic carbon, 1.32% nitrogen, 0.001% phosphorus, and 0.0112% potassium.

Physio-chemical analysis of germination media before sowing

Results revealed that the addition of vermicompost influences both physical and chemical properties of germinating media compared to controls (To: soil + sand and Tcc: soil + sand + cow dung) (Table 1). It was observed that the pH of germination media was slightly decreased (7.6±0.0 to 7.4±0.0) in all treatments after the addition of vermicompost. However, the pH value in control cow dung (Tcc) was 8.7±0.0. Results revealed that Tcc media was thick, crumpled, and greenish-brown, while To germinating media was compact, hard, less porous, and blackish-brown. On the other hand, all vermicompost germinating media were blackish-brown with high porosity and soft aerated texture. Electrical conductivity in To and Tcc was 2.20 ±0.05 mS/cm and 3.48 ±0.01 mS/cm respectively while slightly reduced in all vermicompost containing germination media (from 2.80±0.02mS/cm to 2.73±0.03 mS/cm), respectively. The average percentage of carbon, nitrogen was increased in VC containing germination media compared to controls. Similarly, average phosphorus noted in 35% was 0.062±0.00% followed by 30% (0.051±0.00%) and 25% (0.051±0.00%). The average potassium contents in 35% were recorded as 0.47±0.05%. On the other hand, 10%, 15%, 20%, 25%, and 30% showed average potassium contents as 0.024±0.00%, 0.051±0.00%, 0.031±0.00%, 0.054±0.00% and 0.533 ±0.00% respectively.

Impact of vermicompost on seed germination

Daily observations were made (for 28 days) on the emergence of radicles to analyze the impact of vermicompost on pansy seed germination. Considerable and significant ($p \leq 0.05$ and $p \leq 0.001$) variations among germination parameters at different concentrations of vermicompost were calculated (Table 2). Results revealed that early initiation of seed germination was recorded in 30% VC concentration (3.0±0.0 days) compared to other germination media (Table 2). The maximum mean generation time was recorded in Tcc and To (8.56±0.39 days and 8.10±0.02 days) while the minimum mean generation time was recorded in 30% VC (3.63±0.012 days). The maximum mean daily germination of seeds was found in 30% VC (0.32±0.02 days) and 25% VC (0.30±0.05 days) while the minimum value was found in To (0.21±0.01 days) and Tcc (0.22±0.02 days). The speed of germination was highest at 30% VC (9.63±0.08) and 35% VC (9.31±0.01). The lowest speed of germination was observed in Tcc (5.33±0.08) and To (4.81±0.08). Data revealed that the minimum days were taken for completion of germination in 30% VC (6.33±0.47 days) compared to other germination media compositions (Table 2). The maximum percentage of germination (100.0±0.0 %) was recorded at 30 % VC followed by (96.3±0.47 %) at 25% VC > (91.6±1.24 %) at 20% VC > (81.6 ±0.94 %) at 15% VC > (81.5 ±1.22 %) at 10% VC > (76.0±0.0 %) at 35% VC > (75.6±0.47 %) at Tcc, respectively (Table 2). However, the minimum germination percentage was recorded in To (63.0±0.0 %). The maximum peak value was observed in 30% VC (1.64±0.01) compared to other germination media. The maximum germination value was recorded at 30% VC (0.52±0.01) followed by 20% VC (0.40±0.05) while the minimum germination value was recorded in To (0.11±0.05), in Tcc (0.237±0.0), and at 10% VC (0.21±0.12). The whole results of seed germination indicate that all germination parameters were maximum at 30% VC germination media compared to others. Thus, the current research indicates that 30%VC is the preferable germination media for pansy seeds. The impact of various vermicompost containing germination media and controls (To and Tcc) on pansy seed germination is shown in figure 1.

Seedling growth parameters

A significant ($p \leq 0.001$) increased in all growth parameters were recorded in treated plants when compared to control plants (Figure 1). Results revealed that 30% VC germinating media showed a significant increase in shoot length (3.66±0.47 cm), a number of leaves (7.33±0.47), the diameter of leaves (3.00 ±0.0 cm), length of leaves (3.66±0.47 cm), leaf area (6.33±0.47 cm), whole seedling length (9.00±0.0 cm), and root length (4.66±0.47 cm) of pansy

(Figure 1; Table 3). From the whole data, it was observed that vermicompost has a significant impact on the seedlings of pansy compared to controls (Table 3; Figure 1).

Physio-chemical analysis of germination media after seedling harvesting

After harvesting pansy seedlings, the germinating media was again analyzed to check the remaining nutrients profile. All germination media showed significant ($p \leq 0.001$) decreased values of carbon, nitrogen, phosphorus, and potassium compared to media prepared used for seed sowing (Table 1). However, pH values remain the same such as 8.7±0.0 in Tcc, 7.6±0.0 in To, and 7.4±0.0 in all VC treatments. Electrical conductivity was slightly reduced in To and Tcc (2.17±0.37 mS/cm and 3.38±0.10 mS/cm) respectively. Similarly, EC was also reduced in all VC treatments (2.71±0.01 mS/cm to 2.49±0.05 mS/cm) at $p \leq 0.001$.

Physio-chemical properties of the germinating media before transplantation of seedling

The pH, EC, and nutrient contents (N, P, K, C) in germinating media was analyzed before the transplantation of the seedling (Table 4). The pH was recorded in Tcc and To as 8.7±0.0 and 7.6±0.0 whereas 7.4±0.0 was recorded in all VC treatments. The texture and appearance vary in controls and vermicompost treatments, i.e. Tcc was thick crumpled with greenish-brown color, To was compact, less porous having brownish-black color whereas all vermicompost treatments were blackish-brown with high porosity, and soft aerated. Electrical conductivity in To and Tcc was recorded as 2.13±0.01 mS/cm and 3.45±0.05 mS/cm while a range between 2.84±0.01 mS/cm to 2.97±0.02 mS/cm was recorded in all VC treatments.

Plant growth parameters

The vegetative plant growth parameters (plant height, volume of root, number of shoots per plant, and length of roots and shoots) and floral parameters (No. of flowers, no. of floral buds, the diameter of flower, fresh and dry weight of flowers) showed significant ($p \leq 0.001$) results in case of vermicompost containing germination media compared to controls Tcc and To (Figure 2; Table 5). The maximum plant height (12.33±0.47 cm) was observed in 35% VC germinating media while the minimum height of the plant was obtained in Tcc (3.5±0.40 cm) and To (3.16±0.23 cm). The maximum root volume 7.33±0.47 and 6.66±0.47 were recorded at 35% VC and 25% VC while the minimum root volume was recorded in To (3.66±0.47). The maximum number of leaves per plant was observed at 35% VC (22.3±0.47) and the minimum number of leaves per plant was observed in Tcc (19.00 ±0.81) and To (18.33 ±1.27). Table 5 reveals the minimum average diameter of pansy plants grown in To and Tcc (2.16±0.23 cm and 2.33±0.23 cm), while the maximum average diameter of leaves was recorded in 35% VC (4.16±0.23) cm. A maximum number of open flowers per plant was recorded at 35% VC (3.33±0.47) whereas the minimum number of flowers was recorded in Tcc (1.00±0.0) and To (1.66±0.47). The maximum number of floral buds was observed in 35% VC (3.33±0.22) and the minimum number of floral buds was observed in control Tcc (1.00±0.0)**.**The maximum diameter (7.83±0.23 cm) of pansy flower was observed at 35% VC whereas a minimum diameter (3.66 ±0.47) cm was observed in control Tcc. The maximum fresh weight (13.66±0.47 g) of flowers was observed in 35% VC whereas control To and control Tcc showed a minimum fresh weight of flowers i.e. 2.33±0.23 g and 1.83±0.23 g. The maximum dry weight (7.2±0.08) of flowers was observed in 35% VC whereas To and Tcc showed the minimum dry weight of flowers i.e. 0.3±0.00 g and 0.1±0.00 g respectively. From the whole results, we can say that for the development of plant growth parameters 35% VC germinating media is the best media as shown in figure 2.

Post harvesting physio-chemical analysis of the germinating media

The nutrient contents (N, P, K, C) were again analyzed after harvesting of pansy plants to check the nutrient efficiency of germinating media. The pH value of Tcc and To was recorded as 8.7±0.0 and 7.6±0.0. Similarly, the same pH value 7.4±0.0 were recorded in all VC treatments. The texture and appearance of Tcc was thick, crumpled with greenish brown color, To germinating media was compact, less porous, and hard with brownish-black color, whereas all germinating media having vermicompost were blackish-brown with high porosity and soft aerated texture. Electrical conductivity in To and Tcc slightly decreased (from 2.13±0.01 mS/cm to 2.07±0.09 mS/cm and from 3.45±0.05 mS/cm to 3.34±0.02 mS/cm). Similarly, electrical conductivity in all vermicomposting media was also declined and recorded in range between 2.74±0.01 mS/cm to 2.86±0.05 mS/cm. Germinating media having vermicompost showed significant declined in carbon, nitrogen, phosphorus and potassium when compared to nutrient content of germinating media before transplantation (Table 4).

DISCUSSION

Vermicompost stimulate plant growth due to earthworm involvement in its formation. Various studies were conducted on the utilization of vermicompost on the seed germination and development of plant growth (Atiyeh et

al. 2001a). According to Khan and Ishaq. (2011) vermicompost seems to be the most encouraging bio-fertilizer which not just enhanced the growth of plants and supply of nutrients but also free of pollution and cost-effective. Vermicomposting is a joint action of microorganisms and earthworms in environment-controlled conditions, hence achieving non-thermophilic decomposition and stabilization of organic fraction of solid waste (Deka et al. 2011). In our study vermicompost was prepared under similar nonthermophilic moisture-loving conditions via utilizing *Eisenia fetida*. Enough space for vertical and horizontal movements was provided for earthworms and our resultant product was blackish-brown, light weight, a porous structure having a soft granular appearance. Our findings are thus parallel to the work of Munnoli et al. (2002) who reported that vermicompost has a blackish-brown color, having high porosity, aeration, drainage, and water holding capacity. Our study showed that soil amended with vermicompost had significantly greater soil bulk density and hence porous, lighter, and never compacted.

In the current research, cow dung manure was used for the vermicompost production. Our results are consistent with the findings of Acıkbas and Belliturk. (2016). Vermicomposting is a bio-oxidation, non-thermophilic and aerobic process due to the presence of epigeic earthworms (Edwards and Burrows, 1988). Earthworms have a great impact on soil productivity and fertility (Gunadi et al. 2002). Swati and Hait. (2017) demonstrated that vermicompost is an eco-friendly, cost-effective, and perfect soil improvement fertilizer. Several kinds of literature illustrated the utilization of earthworms increases soil organic matter (Gorres et al. 2016; Belliturk et al. 2017). Dominguez et al. (2010) described that earthworms enhance microbial activity especially about soil quality and crop production. In the current research, *E. fetida* has been used for the vermicompost production. According to the Belliturk. (2016) and Belliturk et al. (2015a), *E. fetida* is the best choice for outdoor vermicomposting. Vermicompost has been produced using goat and sheep manure, raw vegetables, and fruits wastes, tea wastes, paper wastes (Zahmacioglu and Belliturk, 2014), coconut peel, wheat, and rice straws (Acıkbas and Belliturk, 2016). In our study, the temperature and pH of the vermicompost were recorded as 25°C and 7.8. This temperature and pH range is in the optimum range for growing media and our findings are parallel to Mohee and Mudhoo, (2005) who stated that the optimal range of temperature is 22°C to 25 °C and pH from 5.2 to 7.9. Khater, (2015) suggested that moisture content values ranged from 23.5% to 32.10% for different compost types, which is in agreement with our results as the moisture content of our vermicompost was 25%. The electrical conductivity of vermicompost in our study was 2.82mS/cm which is following the findings of Khater, (2015). This EC range is in the optimum range (2.0 to 4.0) for growing media as mentioned by Hanlon, (2012). At the end of the experiment the temperature, pH, EC, and moisture of vermicompost were recorded as 25 °C temperature, 7.8 pH, 2.82 mS/cm EC, and 25 %, respectively. According to Shak et al. (2014), the decrease in EC during vermicomposting may be due to the precipitation or leaching of soluble salts and mineralization of organic acids. On the other hand, physical analysis of cow dung showed that the temperature was 30-45 °C, pH was 8.7, electrical conductivity was 3.52 mS/cm and moisture content was 18% which are in agreement with the work of Khater, (2015) who suggested in his study that temperature, pH and EC of cow manure was 30 °C, 8.1, and 4.1 dSm^{-1}, and moisture content was 20%. EC values below 4.0 mScm−1 isis excellent for agricultural soils (Bhat et al. 2017). Our analysis of vermicompost through atomic absorption spectrophotometry indicated the increased level of trace metals like sulfates, magnesium, manganese, copper, iron, and zinc compared to cow dung, which consistent with the findings of Shahmansouri et al. (2005) who reported the presence of trace metals in vermi-compost. The maximum values of Mg and Ca in reported research were also consistent with the findings of Padmavathiamma et al. (2008). C: N ratio is also very important parameter for the stability and maturity of vermi-compost. Our results agreed with the previous literature (Suthar 2008; Garg and Gupta 2006; Singh et al. 2014). The loss of carbon and nitrogen ratio (11:1) is due to the mineralization process and consistent with the findings of Suthar and Singh (2008) and Shak et al. (2014). They reported that C: N less than 12 is preferable for agricultural purposes.

One of the most important things is that physicochemical changes in vermicompost increased the availability of nutrients to plants, which occurs due to the activity of earthworms and microorganisms, and the mineralization of organic materials (Hane and Pilva, 2013; Juarez, 2011). Vermicompost also has a positive influence on vegetative growth, stimulating shoot growth, and root development (Edwards et al. 2004). Another positive influence of vermicompost application includes the alterations in the morphology of crop plants such as increased leaf area and root branching (Lazcano et al. 2009), stimulating flowering, and increase in the number and biomass of flowers (Arancon et al. 2008; Atiyeh et al. 2002). Vermicompost promotes root initiation, increases root biomass, enhances plant growth, and alters overall plant morphology (Grapelli *et al.* 1987). Our results agreed with the previous literature (Lazcano et al. 2009; Arancon et al. 2008; Atiyeh et al. 2002; Grapelli et al. 1987).

Our study revealed that vermicompost was a perfect nutrient amendment to promote the growth of pansy. In this regard, our results demonstrated that all germination parameters of pansy i.e. early initiation of seed

germination (3 days), minimum mean germination time (3.63±0.0124), maximum mean daily germination (0.32±0.020), highest speed of germination (9.63±0.081), the maximum percentage of germination (100±0.00), highest peak value (1.64±0.012) and maximum germination value (0.52±0.008) was recorded at 30% VC. Similar findings were disclosed by Wilson and Carlile, (1989). According to them, VC showed duality in behavior i.e. increased germination success up to certain concentrations and inhibition at higher levels. They found that duck waste VC in the range 20–30% increased the germination success of tomatoes (*S. lycopersicum*), lettuce (*L. sativa*), and pepper (*Capsicum* sp.), while 50–60% VC treatments reduced the germination success significantly. In a recent series of extensive investigations, it was found that VC derived from salvinia (*Salvinia molesta*), lantana (*Lantana camara*), parthenium (*Parthenium hysterophorus*), and ipomoea (*Ipomoea carnea)* enhanced the germination success of lady's finger, green gram, and cucumber when used in the concentration range of 25–35% but hindered it at higher levels of application (Hussain et al. 2015, Hussain et al. 2016, Hussain et al. 2017 and Hussain et al. 2018.) Based on our observations regarding seed germination, it is expected that water-soluble bioactive substances such as humic acids, phytohormones, or other microbial metabolites present in vermicompost could be responsible for earlier emergence, increased seed germination percentage, and seedling growths and consistent with the Arancon et al. (2007) and Lazcano et al. (2010).

Although seed germination is an internally regulated process influenced by genotype, external factors such as light, temperature, moisture, and the presence of certain chemical compounds (phytohormones or organic acids) also strongly influence seed germination (Finkelstein, 2004; Kucera et al. 2005). Our study also suggested a significant effect of vermicompost on seedling parameters compared to control treatments. We can say that 30% VC was a preferable dose for seedling growth parameters because highest shoot length (3.66±0.47 cm, maximum number of leaves per plant (7.33±0.47), the maximum diameter of leaves (3.00 ±0.00 cm), the maximum length of leaves (3.66±0.47 cm), maximum leaf area (6.33±0.47 cm), maximum seedling length (9.00±0.00 cm) and root length (4.66±0.47 cm) were recorded at this concentration. Arancon et al. (2003) and Arancon et al. (2005) suggested that seedling growth was better in the VC amended media, compared to the control media, even when all the nutrients equivalent to those contained in the VC were given to the plants growing in the control media. They postulated that it was the plant growth hormones and humic acids in the VC that, in conjunction with better nutrient availability, exerted the beneficial effect.

Current research indicated the maximum vegetative and reproductive growth of pansy at 35% VC compared to controls (Sand + Soil + cow dung and Sand + Soil). As compared to other fertilizers, pansies cultivated in a slow-release fertilizer showed the most abundant flowering (Zawadzinska and Janicka, 2007). They found that the pansies cultivated in the medium containing 75% of compost from sewage sludge, potato pulp, and sawdust produced the largest flowers of the greatest width, the longest with the longest flower stalk, and our work also refers to the point that higher doses of slow-release fertilizer like vermicompost promotes overall growth pattern in plants. In the current research, the maximum vegetative growth parameters such as maximum plant height (12.33±0.47 cm), root volume (7.33±0.47 cm^3), number of leaves per plant (22.3±0.47) and diameter of leaves (4.16±0.23 cm), and flowering characteristics like maximum number of floral buds per plant (3.33 ±0.47), number of open flowers per plant (3.33±0.47), the diameter of flowers (7.83 ±0.23) were obtained at 35% VC. Similarly, at 35% VC initiation of floral buds after transplantation of plants occurs earlier at 74.6 days compared to control cow dung (81.4 days) and control soil (87.6 days). Our studies are thus parallel to the findings of Atiyeh *et al.* (2002) and Arancon *et al.* (2008) who studied the effects of vermicompost incorporation in the potting media of two ornamental plants, marigolds, and petunias and reported that vermicompost increased significantly the growth of both species and that vermicompost incorporation up to 40% produced significant increases vegetative growth as well as in the number of flowers per plant, the most important feature in the production of ornamentals. However, our findings are contrary to that of work of the Lazcano and Dominguez, (2010), who reported that the highest dose of vermicompost produced a decrease in the survival rate of the pansy plants. They concluded that the addition of 25% vermicompost to the potting media of pansies reduced significantly the number of flowers produced, flower biomass independently of the type of vermicompost considered.

Our results demonstrate that post harvested germinated media contain significantly few bio-nutrients compared to the media used before transplantation and sowing. This may be because the plant grows it extracts nutrients from the germinating media, and nutrients move up in the plant body thereby reducing the nutrient content in germinating media and also because the bioavailability of nutrients in vermicompost is more compared to cow dung and synthetic fertilizers, thus all vermicompost treatments showed significantly few nutrients after harvesting of pansy plants. Our results reveal that maximum germination of pansy seedlings occurred at 30% VC concentration, more nutrients are up taken by plants body, that's why 30% VC germinating media showed the least amount of nutrient

profile compared to other vermicompost concentrations. Maximum seedling growth also occurred at 30% VC as at this concentration more nutrients are extracted from germinating media and moved up in the plant body. Similarly, in a 7 kg potting mixture maximum decline of nutrients is shown by 35% VC post harvested media, ensuring the maximum uptake of nutrients in the plant body resulting in maximum vegetative and reproductive characteristics (Figure 2). Nutrient analysis of both controls Tcc and T_o suggested maximum retention of nutrients in media, thus allowing minimum nutrients to become bioavailable and up taken by plants resulting in sluggish germination as well as overall growth.

Conclusion

In the current research, the impact of organic manure and vermicompost on the seed germination, seedlings, and growth parameters of pansy were comparatively studied. Seed germination and seedling parameters were maximum at 30% VC compared to other treatments. Based on plant valuation scale, quality analysis, and abundance of flowering, the germinating media containing 35% vermicompost supplement are found to be the best for pansy cultivation. The whole results reveal the potent effect of vermicompost on pansy compared to the control treatments. We suggested that appropriate dose for nursery raising of pansy plants should be 30% VC whereas 35% VC is appropriate for the maximum vegetative growth, reproductive growth, and flowering of marigold. Therefore, vermicompost could be used as a natural bio-fertilizer to increase ornamental plant's production and for a sustainable horticulture system instead of inorganic fertilizer. The expenditure on costly chemical fertilizer input may also be reduced by applying vermicompost for ornamental plant's production.

REFERENCES

Tilman D, Cassman KG, Matson PA, Naylor R Polasky S. Agricultural sustainability and intensive production practices. Nature. 2002; 418: 671-677.

Hutchison ML, Walters LD, Avery SM, Munro F, Moore A. Analyses of livestock production, waste storage, and pathogen levels and prevalence in farm manures. Appli Environm Microbiol. 2005; 71: 1231–1236.

Lazcano C, Gomez-Brandon M, a Dominguez J. Comparison of the effectiveness of composting and vermicomposting for the biological stabilization of cattle manure. Chemosphere. 2008; 72: 1013-1019.

Edwards CA, Dominguez J, Arancon NQ. The influence of vermicomposts on plant growth and pest incidence. *In:* Soil Zoology for Sustainable Development in the 21st Century (Shakir, S.H., Mikhaïl, W.Z.A., Eds.), Cairo, pp. 397-420. 2004.

Lim SL, Lee LH, Wu TY. Sustainability of using composting and vermicomposting technologies for organic solid waste biotransformation: recent overview, greenhouse gases emissions and economic analysis. J Clean Prod. 2016; 111: 262–78.

Bhat SA, Singh J, Vig AP. Vermiremediation of dyeing sludge from textile mill with the help of exotic earthworm Eisenia fetida Savigny. Environ Sci Pollut Res. 2013; 20: 5975–82.

Ostos JC, Lopez-Garrido R, Murillo JM, Lopez R. Substitution of peat for municipal solid waste and sewage sludge based composts in nursery growing media: effect on growth and nutrition of the native shrub Pistacia lentiscus. Bioresour Technol. 2008; 99: 1793–800.

Bhat SA, Bhatti SS, Singh J, Sambyal V, Nagpal A, Vig AP. Vermiremediation and phytoremediation: eco approaches for soil stabilization. Austin Environ Sci. 2016a; 1:1006-1012.

Rajendran M, Thivyatharsan R. Performance of different species of earthworm on vermicomposting. Inter J Res. 2014; 2: 2311-2476

Garcia-Gomez A, Bernal MP, Roig A. Ornamental plants growth in substrates using composts from agroindustrial wastes. BioresTechnol. 2002; 83: 81–87.

Chaoui HI, Zibilske LM, Ohno T. Effects of earthworm casts and compost on soil microbial activity and plant nutrient availability. Soil Biol Biochem. 2003; 35: 295-302

Edwards CA, Burrows I. The potential of earthworm composts as plant growth media. In Earthworms in Environmental and Waste Management Ed. C. A., Neuhauser, SPB Academic Publ. b.v. The Netherlands. 1988. 211-220.

Orozco SH, Cegarra J, Trujillo LM, Roig A. Vermicomposting of coffee pulp using the earthworm Eisenia fetida: effects on C and N contents and the availability of nutrients. Biol Fertil Soil. 1996; 22: 162-166.

Marfa O, Lemaire F, Caceres R, Giuffrida F, Guerin V. Relationships between growing media fertility, percolate composition and fertirrigation strategy in peat-substitute substrates used for growing ornamental shrubs. Sci Hortic- Amsterdam. 2002; 94: 309-321.

Startek L 2003 Cultivation facilities and decorative value of garden pansy. Rosliny Ozdobne, 2: 16-18.

Bohdanowicz KE, Jerzy, Małobęcki, Andrzej, Aneta S. "Floral and pollen characters a useful tools in *Viola* taxonomy" (PDF). Acta Biologica Cracoviensia. 2012; 54: 18-29.

Lewis WH, Elvin-Lewis MPF. *Medical Botany. Plants Affecting Human Health* (p.555). Hoboken: John Wiley & Sons. 2003

Zawadzinska A, Janicka D. Effects of compost media on growth and flowering of parviflorous garden Pansy (*viola wittrockiana* gams) part ii. plant flowering and decorative value, Acta Agrobotanica. 2007; 60: 167–171.

Startek L, Janicka D, Dobrowolska A, Placek M. The effects of medium on emergence dynamics and the length of vegetative stage of pansy belonging to Carrera and Butterfl y groups. Zesz. Prob. Post. Nauk Rol. 2006a; 510: 601-608.

Bhatnagar RK, Palta R. Earthworm Vermiculture and Vermicomposting. Kalyani Publishers, Ludhiana, India.1996-

Apha, Awwa, Wef. Standard methods for water and wastewater examinations. Washington DC, 22th editions, 2012-

Watanabe FS, Olsen SR. Test of an ascorbic acid method for determining phosphorus in water and NaHCO3 extracts from soil. Soil Sci Soc Am Proc. 1965; 29: 677 – 678.

Olsen SR, Sommers LE. *Phosphorus*. p. 403 – 430. *In* A. L. Page (ed.), *Methods of soil analysis, Agron. No. 9, Part 2: Chemical and microbiological properties,* 2nd ed., Am. Soc. Agron., Madison, WI, USA. 1982.

Naouni F, Protano G, Riccobono F. Uptake & bioaccumulation of heavy elements by two earthworm species from a smelter contaminated area in northern Kosovo. Soil Biol Biochem. 2011; 43: 2359–67.

Shahmansouri M, Pourmoghadas H, Parvaresh A, Alidadi H. Heavy metals bioaccumulation by Iranian and Australian earthworms (*Eisenia fetida*) in the sewage sludge vermicomposting. Iran J Environ Heal Sci & Engin.2005; 2: 28–32.

Czabator FJ. Germination Value: An Index Combining Speed and Completeness of Pine Seed Germination. Forest Science. 1962; 8:386-396

Ellis R, Roberts E. Towards a rational basis for testing seed quality. Butter worths, London. 1981

ISTA. International Rules for Seed Testing. Seed Sci Technol 27, Supplement. 1999.

Atiyeh RM, Arancon N, Edwards CA, Metzger JD. The influence of earthworm-processed pig manure on the growth and productivity of marigolds, Biores Technol. 2001a; 81: 103-108.

Khan A, Ishaq F. Chemical Nutrient Analysis of different Composts Vermicompost and Pit Compost) and their Effect on Growth of a Vegetative Crop (*Pisum sativum*). Asian J Plant Science Res. 2011; 1: 116-130.

Deka H, Deka S, Baruah CK, Das J, Hoque S, Sarma NS. Vermicomposting of distillation waste of citronella plant (Cymbopogon winterianus Jowitt.) employing *Eudrilus eugeniae*, Biores Technol. 2011; 102: 6944–6950.

Munnoli PM, Arora JK, Sharma SK. Impact of vermi-processing (by earthworms) on soil characteristics. J Ind Pollut Contr. 2002; 18: 87 – 92

Acıkbas B, Belliturk K. Vermikompostun Trakya İlkeren/5BB Aşı Kombinasyonundaki Asma Fidanlarının Bitki Besin Elementi İçerikleri Üzerine Etkisi. J Tekird Agri Facul. 2016a; 13:131-138.

Gunadi B, Edwards CA, Arancon NQ. Changes in trophic structure of soil arthropods after the application of vermicomposts. Eu J Soil Biol. 2002; 38: 161-165.

Swati A, Hait S. Fate and bioavailability of heavy metals during vermicomposting of various organic wastes—A review. Proc Safe Environ Protect. 2017a; 109: 30-45.

Gorres JH, Belliturk K, Melnichuk RD. Temperature and moisture variables affecting the earthworms of genus Amynthas Kinberg, 1867 (Oligachaeta: Megascolecidae) in a hardwood forest in the Champlain Valley, Vermont, USA. Appl Soil Ecol. 2016; 104:111-115.

Belliturk K, Adiloglu S, Solmaz Y, Zahmacıoglu A, Adiloglu A. Effects of Increasing Doses of Vermicompost Applications on Phosphorus and Potassium Concentrations of Pepper (Capsicum annuum L.) and Eggplant (Solanum melongena L.). J Adv Agri Technol. 2017; 4: 372-375.

Dominguez, Dominguez J, Aira M, Gomez-Brandon M. Vermicomposting: Earthworms Enhance the Work of Microbes, In: Microbes At Work: From Wastes to Resources, H. Insam; I. Franke-Whittle; M. Goberna, (Eds.), 93-114, Springer, ISBN 978-3-642- 04042-9, Heidelberg, Germany. 2010

Belliturk K. Vermicomposting Technology for Solid Waste Management in Sustainable Agricultural Production. Cukurova J Agric Food Sci. 2016; 31: 1-5.

Belliturk K, Gorres JH, Kunkle J, Melnichuk RD. Can commercial mulches be reservoirs of invasive earthworms? Promotion of ligninolytic enzyme activity and survival of Amynthas agrestis (Goto and Hatai, 1899). Applied Soil Ecol. 2015a; 87: 27-31.

Zahmacıoglu A, Belliturk K. Evaluation of Paper Wastes by Vermicomposting. Tubitak 2209/A, Support Program of University Student Research Project, No: 1919B011303253, Ankara. 2014

Mohee R, Mudhoo A. Analysis of the physical properties of an invessel composting matrix. Powd Technol. 2005; 155: 92–99.

Khater ESG. Some Physical and Chemical Properties of Compost. Inter J Waste Resour. 2015; 5: 1-5.

Hanlon EA. Soil pH and electrical conductivity: a count extension soil laboratory manual. IFAS Extension, University of Florida, FL, USA.2012

Shak KPY, Wu TY, Lim SL, Lee CA. Sustainable reuse of rice residues as feedstocks in vermicomposting for organic fertilizer production. Environ Sci Pollut Res. 2014; 21: 1349–1359

.Bhat SA, Singh J, Vig AP.Instrumental characterization of organic wastes for evaluation of vermicompost maturity. J Analyti Sci Technol. 2017; 8: 2-12.

Padmavathiamma K, Loretta Y, Usha LI, Kumari. An experimental study of vermi biowaste composting for agricultural soil improvement. Bioresour Technol. 2008; 99:1672–1681

Suthar S. Microbial decomposition efficiencies of monoculture and polyculture vermireactors based on epigic and anecic earthworms. World J Microbial Technol. 2008; 24: 1471-1479

Garg P, Gupta A, Satya S. Vermicomposting of different types of waste using *Eisenia fetida*: a comparative study. Bioresour Technol.2006; 97:391–395

Singh A, Singh RV, Saxcena AK, Shivay YS, Nain L. Comparative studies on composting efficiency of *Eisenia fetida* and *Perionyx excavatus*. J Experi Bioland Agricul Sci. 2014; 2: 508–17.

Suthar S, Singh S. Feasibility of vermicomposting in biostabilization of sludge from a distillery industry. Sci Total Environ. 2008; 394: 237–43.

Hane A, Pilva P. Vermicomposting technology as a tool for nutrient recovery from kitchen biowaste. J Materi Cycl Waste Manage. 2013; 13: 431–9.

Juarez PDA, Fuenta JL, Paulin RV. Vermicomposting as a process to stabilize organic waste and sewage as an application for soil. Trop Subtrop Agroecosys. 2011; 14: 949–63.

Lazcano C, Arnold J, Tato A, Zaller JG, Dominguez J. Compost and vermi-compost as nursery pot components: Effects on tomato plant growth and morphology, Span J Agri Res. 2009; 7: 944-951.

Arancon NQ, Edwards CA, Babenko A, Cannon J, Galvis P, Metzger JD. Influences of vermicomposts, produced by earthworms and microorganisms from cattle manure, food waste and paper waste, on the germination, growth and flowering of petunias in the greenhouse, Appl Soil Ecol. 2008; 39: 91-99.

Grappelli A, Galli E, Tomati U. Earthworm casting effect on *Agaricus bisporus* fructification. Agrochimica. 1987; 21: 457-462.

Wilson DP, Carlile WR. Plant growth in potting media containing worm-worked duck waste. Acta Horticult, 1989; 238: 205–220

Hussain N, Abbasi T, Abbasi SA. Vermicomposting eliminates the toxicity of lantana (*Lantana camara*) and turns it into a plant friendly organic fertilizer. J Hazard Mater. 2015; 46–57.

Hussain N, Abbasi T, Abbasi SA. Vermicomposting transforms allelopathic parthenium into a benign organic fertilizer. J Environ Manage. 2016; 180: 180–189.

Hussain N, Abbasi T, Abbasi SA. Toxic and allelopathic ipomoea yields plant-friendly organic fertilizer. J Clean Product. 2017; 148: 826–835.

Hussain N, Abbasi T, Abbasi SA. Generation of highly potent organic fertilizer from pernicious aquatic weed *Salvinia molesta*. Environ Sci Pollut Res. 2018; 25: 4989–5002

Arancon NQ, Edwards CA, Dick, Dick L. Vermi-compost tea production and plant growth impacts. Biocycle.2007; 48: 51–52.

Lazcano C, Dominguez J. Effects of vermi-compost as a potting amendment of two commercially-grown ornamental plant species. Span Agri Res. 2010; 8: 1260-1270

Finkelstein RR. Hormones in seed development and germination, In: Davies, P .J. (ed.). Plant hormones: Biosynthesis, signal transduction and action, Kluwer Academic Publishers p. 2004; 513–537.

Kucera B, Cohn MA, Leubner-Metzger G. Plant hormone interactions during seed dormancy release and germination, Seed Science Res. 2005; 15: 281–307.

Arancon NQ, Edwards CA, Bierman P, Metzger JD, Lee S, Welch C. Effects of vermicomposts on growth and marketable fruits of field-grown tomatoes, peppers and strawberries, Pedobiologia, 2003; 47: 731–735.

Arancon NQ, Edwards CA, Bierman P, Metzger JD, Lucht C. Effects of vermicomposts produced from cattle manure, food waste and paper waste on the growth and yield of peppers in the field. Pedobiologia. 2005; 45: 634-641.

Figure 1. Impact of germinating media on seed germination of pansy.

Figure 2. Impact of germinating media on plant growth parameters at 15th week of transplantation.

Table 1. Pre and post physio-chemical analysis of germinating media used for pansy seed germination and seedling growth parameter

Parameters→ Treatments ↓		pH	Appearance	EC	C (%)	N (%)	K (%)	P (%)
**Control Tcc**	**Pre**	8.7±0.0	Thick crumpled, greenish brown	3.48 ±0.01	0.80±0.05	0.83±0.05	0.01±0.00	0.038±0.00
	**Post**	8.7±0.0	Thick crumpled greenish brown	3.38±0.10	0.61±0.01	0.66±0.01	0.01±0.00	0.029±0.00
**Control To**	**Pre**	7.6±0.0	Blackish brown, compact, less porous	2.20±0.05	0.97±0.01	0.83±0.05	0.01±0.00	0.038±0.00
	**Post**	7.6±0.0	Blackish brown, compact, less porous	2.17±0.37	0.82±0.01	0.66±0.05	0.01±0.00	0.018±0.00
**10% VC**	**Pre**	7.4±0.0^{aaa,bbb}	Blackish brown, porous	2.80 ±0.02^{aaa,bbb}	1.30±0.05^{aaa,bbb}	1.01±0.01^{aaa,bbb}	0.02±0.00^{aaa,bbb}	0.041±0.00^{aaa,bbb}
	**Post**	7.4±0.0^{aaa,bbb}	Blackish brown, porous	2.71±0.01^{aaa,bbb}	1.14±0.01^{aaa,bbb}	0.90±0.01^{aaa,bbb}	0.02±0.00^{aaa,bbb}	0.024±0.00^{aa,bbb}
**15% VC**	**Pre**	7.4±0.0^{aaa,bbb}	Blackish brown, porous	2.77±0.02^{aaa,bbb}	1.34±0.01^{aaa,bbb}	1.13±0.05^{aaa,bbb}	0.051±0.00^{aaa,bbb}	0.044±0.00^{aaa,bbb}
	**Post**	7.4±0.0^{aaa,bbb}	Blackish brown, porous	2.72±0.02^{aaa,bbb}	1.21±0.01^{aaa,bbb}	0.94±0.01^{aaa,bbb}	0.040±0.00^{aaa,bbb}	0.035±0.00^{aaa,bbb}
**20% VC**	**Pre**	7.4±0.0^{aaa,bbb}	Blackish brown, porous	2.73±0.03^{aaa,bbb}	1.44±0.01^{aaa,bbb}	0.57±0.05^{aaa,bbb}	0.03±0.00^{aaa,bbb}	0.026±0.00^{aaa,bbb}
	**Post**	7.4±0.0^{aaa,bbb}	Blackish brown, porous	2.59 ±0.01^{aaa,bbb}	1.24±0.05^{aaa,bbb}	0.33±0.01^{aaa,bbb}	0.022±0.00^{aaa,bbb}	0.018±0.00^{aaa,bbb}
**25% VC**	**Pre**	7.4±0.0^{aaa,bbb}	Blackish brown, porous	2.73±0.03^{aaa,bbb}	1.40±0.05^{aaa,bbb}	1.01±0.01^{aaa,bbb}	0.051±0.00^{aaa,bbb}	0.054±0.00^{aaa,bbb}
	**Post**	7.4±0.0^{aaa,bbb}	Blackish brown, porous	2.54±0.05^{aaa,bbb}	1.18±0.01^{aaa,bbb}	0.89±0.01^{aaa,bbb}	0.04±0.00^{aaa,bbb}	0.031±0.00^{aaa,bbb}
**30% VC**	**Pre**	7.4±0.0^{aaa,bbb}	Blackish brown, porous	2.73±0.03^{aaa,bbb}	1.51±0.01^{aaa,bbb}	1.23±0.05^{aaa,bbb}	0.533±0.00^{aaa,bbb}	0.051±0.00^{aaa,bbb}
	**Post**	7.4±0.0^{aaa,bbb}	Blackish brown, porous	2.49±0.05^{aaa,bbb}	1.32±0.01^{aaa,bbb}	0.3±0.01^{aaa,bbb}	0.01±0.00^{aaa,bbb}	0.014±0.00^{aaa,bbb}
**35% VC**	**Pre**	7.4±0.0^{aaa,bbb}	Blackish brown, porous	2.73±0.03^{aaa,bbb}	1.35±0.05^{aaa,bbb}	0.94±0.01^{aaa,bbb}	0. 47±0.05^{aaa,bbb}	0.062±0.00^{aaa,bbb}
	**Post**	7.4±0.0^{aaa,bbb}	Blackish brown, porous	2.49±0.05^{aaa,bbb}	1.17±0.02^{aaa,bbb}	0.53±0.01^{aaa,bbb}	0.030±0.00^{aaa,bbb}	0.034±0.00^{aaa,bbb}

Statistical icons: aaa, =p≤0.001

Table. 2. Seed germination parameters in pansy under different compositions of germination media.

Germination parameters → Treatment ↓	Initiation of germination (Days)	Mean germination time (MGT)	Mean daily germination (MDG)	Speed of germination	Completion of germination (Days)	Germination percentage (%)	Peak value	Germination value
**Control Tcc**	6.66±0.47	8.56±0.39	0.22±0.02	5.33±0.08	8.00±0.0	75.6±0.47	0.93±0.01	0.237±0.0
**Control T_0**	6.66±0.47	8.10±0.02	0.21±0.01	4.81±0.08	8.66±0.47	63.0±0.47	0.52±0.01	0.11±0.05
**10% VC**	4.33±0.47^{aaa,bbb}	7.54±0.03^{aaa,b}	0.25±0.01^{a,b}	7.83±0.09^{aaa,bbb}	8.33±0.47	81.5±1.22^{aaa,bbb}	0.83±0.01^{aaa,bbb}	0.21±0.12^{aaa,bbb}
**15% VC**	4.33±0.47^{aaa,bbb}	7.02±0.02^{aaa,bbb}	0.24±0.02^{a,b}	8.10±0.0^{aaa,bbb}	7.66±0.47	81.6±0.94^{aaa,bbb}	0.93±0.01^{aaa,bbb}	0.263±0.05^{aaa,bbb}
**20% VC**	4.00±0.0^{aaa,bbb}	6.50±0.05^{aaa,bbb}	0.31±0.02^{aaa,bbb}	8.48±0.01^{aaa,bbb}	7.00±0.0^{aaa,bbb}	91.6±1.24^{aaa,bbb}	1.33±0.0^{aaa,bbb}	0.40±0.05^{aaa,bbb}
**25% VC**	3.33±0.47^{aaa,bbb}	4.22±0.0^{aaa,bbb}	0.30±0.05^{aaa,bbb}	9.12±0.02^{aaa,bbb}	8.33±0.47	96.3±0.47^{aaa,bbb}	1.02±0.01^{aaa,bbb}	0.31±0.05^{aaa,bbb}
**30% VC**	3.00±0.0^{aaa,bbb}	3.63±0.01^{aaa,bbb}	0.32±0.02^{aaa,bbb}	9.63±0.08^{aaa,bbb}	6.33±0.47	100±0.0^{aaa,bbb}	1.64±0.01^{aaa,bbb}	0.52±0.01^{aaa,bbb}
**35% VC**	4.33±0.47^{aaa,bbb}	4.16±0.01^{aaa,bbb}	0.25±0.00^{a,b}	9.31±0.01^{aaa,bbb}	8.33±0.47	76.0±0.0^{aaa,bbb}	0.85±0.00^{aaa,bbb}	0.212±0.0^{aaa,bbb}

Statistical icons: a =p≤0.05 and aaa, =p≤0.001

Table 3. Pansy seedling growth parameters.

Seedling growth parameters → Treatment ↓	Length of shoot (cm)	Total no. of leaves per plant	Diameter of leaves (cm)	Length of leaves (cm)	Leaf area (Lx B x K) (cm)	Whole Seedling length (cm)	Root length (cm)
**Control T_0**	1.66±0.47	4.33±0.47	2.0 ±0.0	1.16±0.23	1.53±0.37	3.66±0.47	1.66±0.47
**Control Tcc**	1.66±0.47	5.33±0.47	2.0±0.0	1.66±0.47	2.26±0.18	5.33±0.47	2.66 ±0.47
**10% VC**	2.33±0.47	4.66±0.47	2.0±0.0	2.16±0.23	2.5±0.40	4.66±0.47	2.66±0.47
**15% VC**	2.33±0.47	5.66±0.47	2.0±0.0	2.33±0.47	2.5±0.75	4.33±0.47	2.66±0.47
**20% VC**	2.66±0.47	6.0±0.0	2.16±0.23	2.33±0.47	3.53±0.40	4.33±0.47	2.66±0.47
**25% VC**	3.33±0.47	6.66±0.47	2.16 ±0.23	3.5±0. 40^{aaa,bbb}	4.0±0.0	7.00±0.0^{aaa,bbb}	3.66±0.47^{aaa,bbb}
**30% VC**	3.66±0.47^{aaa,bbb}	7.33±0.47^{aaa,bbb}	3.0 ±0.0^{aaa,bbb}	3.66±0.47^{aaa,bbb}	6.33±0.47^{aaa,bbb}	9.00±0.0^{aaa,bbb}	4.66±0.47^{aaa,bbb}
**35% VC**	2.66±0.47	6.33±0.47	1.83±0.23	2.33±0.47	3.83±0.23	6.33±0.47	3.00±0.0

Statistical icons: aaa, =p≤0.001

Table 4. Pre and post physio-chemical analysis of germinating media used for pansy vegetative growth parameters

Parameters→ Treatments ↓		pH	Appearance	EC	C (%)	N (%)	K (%)	P (%)
**Control Tcc**	**Pre**	8.7±0.0	Thick crumpled greenish brown	3.45±0.05	0.83±0.23	0.10±0.05	0.0071±0.00	0.040±0.00
	**Post**	8.7±0.0	Thick crumpled, greenish brown	3.34±0.02	0.5±0.08	0.07±0.05	0.0065±0.00	0.034±0.00
**Control To**	**Pre**	7.6±0.0	Blackish brown, compact, less porous	2.13±0.01	0.86±0.05	0.10±0.05	0.0074±0.00	0.040±0.00
	**Post**	7.6±0.0	Blackish brown, compact, less porous	2.07±0.09	0.5±0.05	0.06±0.05	0.0062±0.00	0.032±0.00
**10% VC**	**Pre**	7.4±0.0^{aaa,bbb}	Blackish brown, porous	2.84±0.01^{aaa,bbb}	0.73±0.05^{a,b}	0.066±0.00^{aaa,bbb}	0.021±0.00^{aaa,bbb}	0.039±0.00^{aaa,bbb}
	**Post**	7.4±0.0^{aaa,bbb}	Blackish brown, porous	2.74±0.01^{aaa,bbb}	0.46±0.05^{aaa,b}	0.052±0.00^{aaa,bbb}	0.021±0.00^{aaa,bbb}	0.031±0.00^{aaa,bbb}
15% VC	**Pre**	7.4±0.0^{aaa,bbb}	Blackish brown, porous	2.80±0.00^{aaa,bbb}	0.73±0.05^{a,b}	0.066±0.00^{aaa,bbb}	0.044±0.00^{aaa,bbb}	0.036±0.00^{aaa,bbb}
	**Post**	7.4±0.0^{aaa,bbb}	Blackish brown, porous	2.70±0.05^{aaa,bbb}	0.6±0.08^{aaa,b}	0.055±0.00^{aaa,bbb}	0.035±0.00^{aaa,bbb}	0.028±0.00^{aaa,bbb}
**20% VC**	**Pre**	7.4±0.0^{aaa,bbb}	Blackish brown, porous	2.84±0.02^{aaa,bbb}	1.23±0.05^{aaa,bbb}	0.10±0.00^{aaa,bbb}	0.051±0.00^{aaa,bbb}	0.043±0.00^{aaa,bbb}
	**Post**	7.4±0.0^{aaa,bbb}	Blackish brown, porous	2.74±0.01^{aaa,bbb}	0.83±0.05^{aaa,b}	0.07±0.01^{aaa,bbb}	0.042±0.00^{aaa,bbb}	0.032±0.00^{aaa,bbb}
**25% VC**	**Pre**	7.4±0.0^{aaa,bbb}	Blackish brown, porous	2.90±0.01^{aaa,bbb}	1.01±0.01^{aaa,bbb}	0.10±0.00^{aaa,bbb}	0.05±0.00^{aaa,bbb}	0.049±0.00^{aaa,bbb}
	**Post**	7.4±0.0^{aaa,bbb}	Blackish brown, porous	2.80±0.05^{aaa,bbb}	0.63±0.05^{aaa,b}	0.08±0.05^{aaa,bbb}	0.041±0.00^{aaa,bbb}	0.042±0.00^{aaa,bbb}
**30% VC**	**Pre**	7.4±0.0^{aaa,bbb}	Blackish brown, porous	2.92±0.05^{aaa,bbb}	0.76±0.05^{aa,bb}	0.066±0.00^{aaa,bbb}	0.035±0.00^{aaa,bbb}	0.059±0.00^{aaa,bbb}
	**Post**	7.4±0.0^{aaa,bbb}	Blackish brown, porous	2.84±0.01^{aaa,bbb}	0.53±0.05^{aaa,b}	0.052±0.00^{aaa,bbb}	0.055±0.00^{aaa,bbb}	0.026±0.00^{aaa,bbb}
**35% VC**	**Pre**	7.4±0.0^{aaa,bbb}	Blackish brown, porous	2.97±0.02^{aaa,bbb}	0.366±0.05^{a,b}	0.033±0.00^{aaa,bbb}	0.046±0.00^{aaa,bbb}	0.030±0.00^{aa,bb}
	**Post**	7.4±0.0^{aaa,bbb}	Blackish brown, porous	2.86±0.05^{aaa,bbb}	0.23±0.05^{aaa,b}	0.05±0.05^{aaa,bbb}	0.028±0.00^{aaa,bbb}	0.021±0.00^{aaa,bbb}

Statistical icons: a=p≤0.05; aa=p≤0.01; and aaa=p≤0.001

Table 5. Effect of germination media on vegetative plant growth and flowering parameters of pansy.

Growth and flowering parameters → Treatment ↓	Plant height (cm)	Volume of root (cm^3)	No. of leaves per plant	Diameter of leaves (cm)	No. of open flowers per plant	No. of floral buds	Diameter of flower (cm)	Fresh weight of flower /plant (g)	Dry weight of flower /plant (g)
**Control Tcc**	3.5±0.40	4.16±0.62	19.00±0.81	2.33±0.23	1.00±0.0	1.00±0.0	3.66±0.47	1.83±0.23	0.1±0.0
**Control T$_0$**	3.16±0.23	3.66±0.47	18.33±1.27	2.16±0.23	1.66±0.47	1.66±0.47	6.8±0.05	2.33±0.23	0.3±0.0
**10% VC**	11.00±0.8^{aaa,bbb}	5.33±0.47^{aaa,bbb}	21.00±0.81^{aaa,bbb}	3.88±0.23^{aa,bb}	2.00±0.0	2.33±0.47	6.8±0.23^{aaa}	6.33±0.47^{aaa,bbb}	3.73±0.09^{aaa,bbb}
**15% VC**	6.66±0.47^{aaa,bbb}	4.16±0.23	14.00±0.81	2.83±0.25	2.33±0.47	2.66±0.94	5.66±0.47^{aaa}	8.5±0.40^{aaa,bbb}	4.83±0.12^{aaa,bbb}
**20% VC**	10.66±0.47^{aaa,bbb}	5.5±0.40^{aaa,bbb}	13.3±0.47	4.00±0.0^{aaa,bbb}	3.00±0.0^{aaa,bbb}	3.00±0.0^{aaa,bbb}	6.16±0.23^{aaa}	11.6±0.47^{aaa,bbb}	6.00±0.0^{aaa,bbb}
**25% VC**	9.5±0.40^{aaa,bbb}	6.66±0.47^{aaa,bbb}	21.33±1.24^{aaa,bbb}	3.33±0.47	2.66±0.47	3.00±0.0^{aaa,bbb}	6.5±0.40^{aaa}	10.83±0.23^{aaa,bbb}	5.16±0.12^{aaa,bbb}
**30% VC**	10.66±0.47^{aaa,bbb}	4.66±0.47	12.33±0.47	2.5±0.40	2.66±0.47	2.33±0.47	6.66±0.47^{aaa}	10.83±0.62^{aaa,bbb}	5.16±0.23^{aaa,bbb}
**35% VC**	12.33±0.47^{aaa,bbb}	7.33±0.47^{aaa,bbb}	22.3±0.47^{aaa,bbb}	4.16±0.23^{aaa,bbb}	3.33±0.47^{aaa,bbb}	3.33±0.47^{aaa,bbb}	7.83±0.23^{aaa,bbb}	13.66±0.47^{aaa,bbb}	7.2±0.08^{aaa,bbb}

Statistical icons: aaa=p≤0.001

Figure 1

Click here to access/download;Figure;Figure 1.jpg

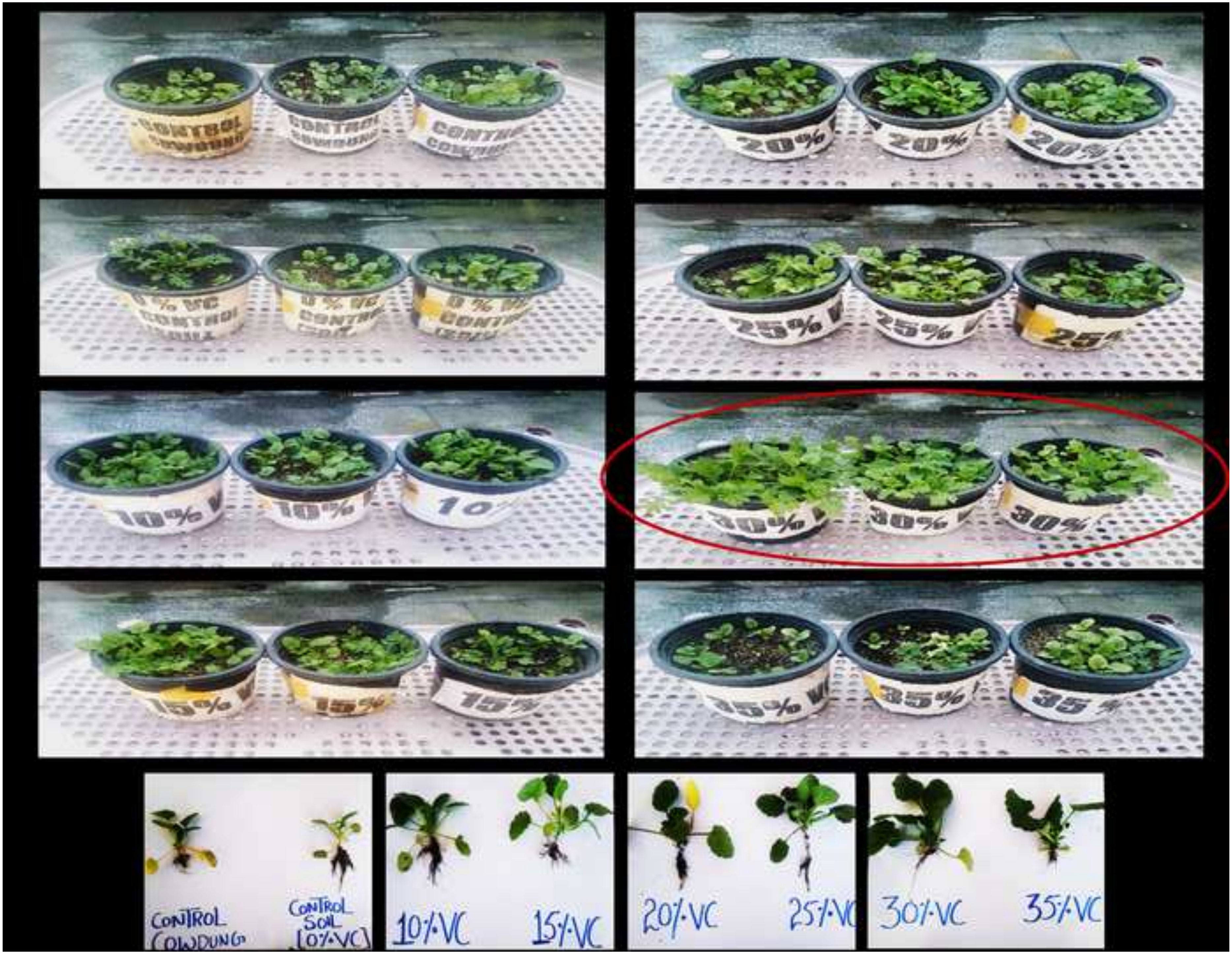

Figure 2

Click here to access/download;Figure;Figure 2.jpg

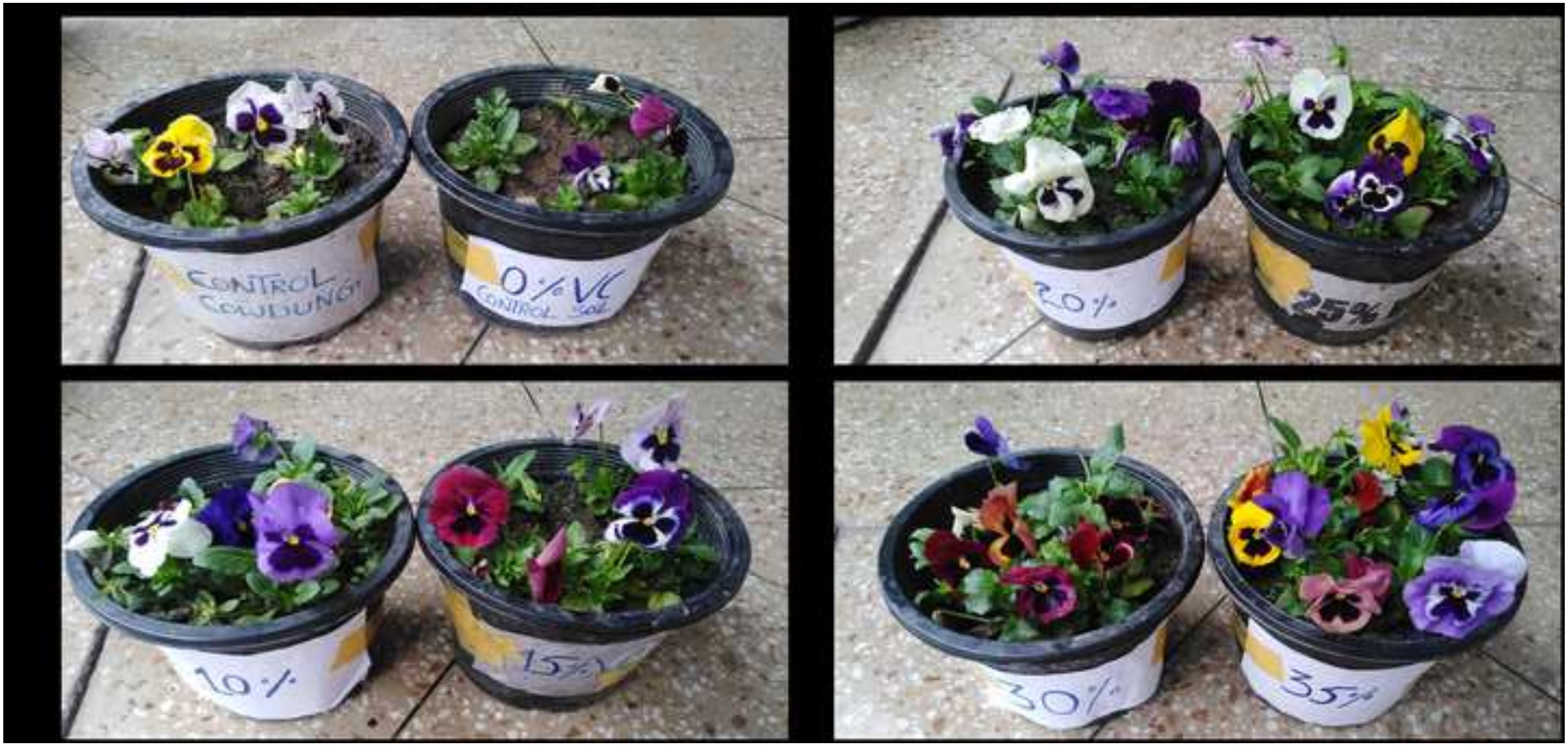

Supplement: Attachment — Submitted filename: PONE-D-22-12324.pdf [file pone.0279828.s002.pdf]
