# Supplementary material for: Cow dung putrefaction via vermicomposting using Eisenia fetida and its influence on seed sprouting and vegetative growth of Viola wittrockiana (pansy)
Source: PLoS One. 2023 Feb 17;18(2):e0279828. doi: 10.1371/journal.pone.0279828 (PMC9937483; doi:10.1371/journal.pone.0279828)
Supplement: Attachment — Submitted filename: response to reviewers.doc [file pone.0279828.s003.doc]

ACADEMIC EDITOR:

This manuscript discusses the impact of vermicompost on seed germination, seedlings, and vegetative growth parameters on plants. There are some serious concerns and points which must be improved. The manuscript fails to identify clearly major achievements in the topic in recent years, major research questions and future research needs. Unfortunately, the information provided in introduction and methodology is insufficient to properly interpret the study. In any case, the Discussion Section must be considerably improved, as it is too general. The manuscript also needs language editing, since it is difficult to understand what the authors are trying to convey in many places. I hope that my comments will help you to improve the quality of this study.

Title: Check the spelling of *Esinea fetida*. It should be *Eisenia fetida*

**Has been corrected**

Abstract: Abstract should be rewritten by detailing the aim and concept of the paper. The abstract should state briefly the purpose of the research, the principal results and major conclusions.

**Has been modified**

Introduction: Very general and need to be elaborative to explore the actual philosophy to design the experiment. The introduction is insufficient to provide the state of the art in the topic. Hypothesis should be given. How this work is different from the available data?

The introduction part needs further improvement. The introduction of the paper must be extended and reformulated in order to provide a more comprehensive approach.

New references are missing in the introduction and discussion. Add 2018-2022 references accordingly

*Objectives of the present study is poorly written. In the last paragraph of the introduction, write the specific objectives of this work clearly, as points (i)..... (ii)....(iii) ......and (iv).

**Introduction has been modified along with recent references.**

Results and Discussion:

The manuscript does not provide interesting and technically sound discussion; it would be better to use more recent references in discussion.  Authors are suggested to add discussion by explaining trends in the obtained results along with the possible mechanisms behind the trends.

Authors are suggested to draw major inferences/primary conclusions followed by the secondary conclusions/ recommendations reached through the critical analysis/ investigation of the study.

**Manuscript has been modified along with recent references.**

5. Review Comments to the Author

Reviewer #1: Dear Authors,

Your manuscript need significant improvement particularly in discussion section to be more compact and reduce the length. Simply delete general or repeated or not necessary sentences and texts.

**Thanks for the suggestions to improve the quality of manuscript. All data have been improved in revised manuscript. Repeated data has been removed.**

Reviewer #2: The manuscript title "Cow dung putrefaction via vermicomposting using Esinea fetida and its impact on seed germination and plant growth parameters of Viola wittrockiana (pansy)" was written very well. However, authors need to address to the review comments for improving the quality of the manuscript prior to the publication process.

**Thanks for the positive recommendations.**
1. Recent references should be incorporated in both introduction and discussion part

**Has been incorporated**
2. Biomass and reproduction data of earthworm is missing.

**Has been incorporated**
3. Reproduction and growth rate should be presented in tabular or graphical form

**Has been presented as figure 1**
4. Grammar and spelling mistakes should be considered/removed in revised manuscript

**Has been removed**
5. conclusion could be improved

**Has been improved**
6. Table 1: Need to mention unit of EC

**Has been mentioned**
7. Table 2: Need to mention the unit of germination speed

**Has been mentioned**
8.Please mention the representation of “bbb” in Statistical icons

**Has been mentioned in statistical analysis section**
