# Supplementary material for: Cow dung putrefaction via vermicomposting using Eisenia fetida and its influence on seed sprouting and vegetative growth of Viola wittrockiana (pansy)
Source: PLoS One. 2023 Feb 17;18(2):e0279828. doi: 10.1371/journal.pone.0279828 (PMC9937483; doi:10.1371/journal.pone.0279828)
Supplement: Attachment — Submitted filename: Response 2.doc [file pone.0279828.s004.doc]

**Response to Reviewers**

Reviewer #3: The authors have addressed the comments and the manuscript has been improved. One minor comment: Please don't use double sign for showing statistical significance of the data. Simply '*' or '***' is enough and generally used in literature. There is no need for other symbols.

**Thanks for the endorsement and recommendation. In the current study authors used two different symbols to represent the differences among the treatments and control groups i.e. ‘*’ represents a significant change among Tcc and VC-treated groups and ‘¥’ represents a significant change among To and VC-treated groups. Otherwise we don’t need to use these symbols.**

Reviewer #4: The authors have addressed the review suggestions to a certain extent; yet the effort is inadequate. Moreover, cow dung itself is a source of bioavailable nutrients because it decomposes quickly. Please provide scientific explanation, why putrefaction of cow dung is nececcasry.

**Thanks for the comments. You are absolutely right that cow dung is itself is a source of bioavailable nutrients but its decomposition is necessary due to following reasons. Manure of cow/horse/chicken is not only too much hot but also contain too high nitrogen contents which is not possible for plants to tolerate. Both temperature and excess nitrogen burns the plants. Secondly, Excess of methane generation and release of toxic gases pollute the environment.**

Also, the introduction must discuss the recent knowledge on vermicomposting. Some papers which are published in recent years should be dicussed and cited, for example,
"Epigenetic regulations enhance adaptability and valorization efficiency in Eisenia fetida and Eudrilus eugeniae during vermicomposting of textile sludge: Insights on repair mechanisms of metal-induced genetic damage and oxidative stress. Bioresource Technology (Elsevier), 2022, 345: 126493"
"Appraisal of lignocellusoic biomass degrading potential of three earthworm species using vermireactor mediated with spent mushroom substrate: Compost quality, crystallinity, and microbial community structural analysis. Science of the Total Environment, (2019), doi: <https://doi.org/10.1016/j.scitotenv.2019.135215>"
"Application of drum compost and vermicompost to improve soil health, growth, and yield parameters for tomato and cabbage plants. Journal of Environmental Management (Elsevier), (2017), 200: 243-252"
"Vermicompost and farm yard manure improves food quality, anti-oxidant and antibacterial potential of Cajanus cajan (L, Mill sp.) leaves. Journal of the Science of Food and Agriculture (Wiley) (2016) 97: 956–966"

**Thanks for the suggestions. All references have been incorporated in revised manuscript.**
